# Referral patterns of children with poor growth in primary health care

**DOI:** 10.1186/1471-2458-7-77

**Published:** 2007-05-11

**Authors:** Floor K Grote, Wilma Oostdijk, Sabine MPF De Muinck Keizer-Schrama, Friedo W Dekker, Paula van Dommelen, Stef van Buuren, Adry M Lodder-van der Kooij, Paul H Verkerk, Jan Maarten Wit

**Affiliations:** 1 Dept. of Paediatrics, Leiden University Medical Center, Leiden, The Netherlands; 2 Dept. of Paediatrics, Erasmus MC – Sophia Children's Hospital, Rotterdam, The Netherlands; 3 Dept of Clinical Epidemiology, Leiden University Medical Center, Leiden, The Netherlands; 4 Dept. of Statistics, TNO Quality of life, Leiden, The Netherlands; 5 Dept. of Methodology & Statistics, University of Utrecht, The Netherlands; 6 Dept. Child Health Care, Regional Public Health Service Hollands Midden, Leiden, The Netherlands; 7 Dept. of Child Health, TNO Quality of life, Leiden, The Netherlands

## Abstract

**Background:**

To promote early diagnosis and treatment of short stature, consensus meetings were held in the mid nineteen nineties in the Netherlands and the UK. This resulted in guidelines for referral. In this study we evaluate the referral pattern of short stature in primary health care using these guidelines, comparing it with cut-off values mentioned by the WHO.

**Methods:**

Three sets of referral rules were tested on the growth data of a random sample (n = 400) of all children born between 01-01-1985 and 31-12-1988, attending school doctors between 1998 and 2000 in Leiden and Alphen aan den Rijn (the Netherlands): the screening criteria mentioned in the Dutch Consensus Guideline (DCG), those of the UK Consensus Guideline (UKCG) and the cut-off values mentioned in the WHO Global Database on Child growth and Malnutrition.

**Results:**

Application of the DCG would lead to the referral of too many children (almost 80%). The largest part of the referrals is due to the deflection of height, followed by distance to target height and takes primarily place during the first 3 years. The deflection away from the parental height would also lead to too many referrals. In contrast, the UKCG only leads to 0.3% referrals and the WHO-criteria to approximately 10%.

**Conclusion:**

The current Dutch consensus guideline leads to too many referrals, mainly due to the deflection of length during the first 3 years of life. The UKCG leads to far less referrals, but may be relatively insensitive to detect clinically relevant growth disorders like Turner syndrome. New guidelines for growth monitoring are needed, which combine a low percentage of false positive results with a good sensitivity.

## Background

Monitoring children's growth and development is fully integrated in preventive health care programmes throughout the world. In developing countries growth monitoring, implying regular height and weight measurements, is primarily aimed at identifying malnutrition to reduce mortality, whereas in industrialised countries it is mainly used to detect disorders associated with growth failure. The effect of routine growth monitoring in developing countries has recently been questioned [1]. Its efficacy and efficiency in developed countries has hardly been studied, and a recent international inquiry showed that there is considerable variability in growth monitoring practices worldwide [2].

The primary aim of growth monitoring in industrialised countries is to detect growth disorders at an early age, thus the sensitivity (a statistical measure of how well the test correctly identifies a condition) of the screening procedure should be high at a young age. Poor growth can be caused by a great diversity of congenital or acquired conditions, some of which present with additional symptoms and signs. In other conditions, such as Turner syndrome, Growth Hormone Deficiency (GHD) and celiac disease, additional clinical features may be absent. Still, in such disorders early diagnosis and treatment is important, because early treatment has an optimal effect on growth in childhood, as well as on final height, expectedly resulting in a better quality of life. The second aim of growth monitoring is to keep the number of healthy children referred for further investigation at a minimum, meaning that the specificity should be very high.

In order to diminish the uncertainty among health workers in preventive child health care about the auxological criteria (auxology is the scientific study of growth) on which the decision to refer a child for further diagnosis should be based, and the resulting wide variation in the way growth monitoring is carried out, in 1996 a consensus meeting was held in the Netherlands on "Diagnosis of short stature in childhood". At this meeting representatives of general practitioners, well-baby clinic doctors, school doctors, paediatricians and paediatric endocrinologists came to a consensus guideline on referral criteria for aberrant growth [3]. Although the authors had aimed at promoting early diagnosis of aberrant growth as well as at preventing unnecessary referral and interventions, it was shown later that if this guideline would be followed, an unacceptable percentage of healthy children would be referred [4]. In the United Kingdom a similar consensus meeting on diagnosis of short stature was held, resulting in a guideline restricted to aberrant height at the age of 5 years, with assumingly a better specificity, but an unknown sensitivity to detect disorders timely [5,6].

In the present study we wished to 1) study the performance of the Dutch Consensus Guideline (DCG) in a second sample with a wider age range; 2) count the actual referrals for short stature in that region; and 3) investigate the test characteristics of the UK Consensus Guideline (UKCG), WHO guideline and several other referral rules. This study was part of a larger program aimed at producing an evidence-based guideline on growth monitoring, in which we earlier investigated the best auxological criteria to detect Turner syndrome [7] Celiac Disease (van Dommelen et al, submitted), and Cystic Fibrosis (Grote et al, submitted).

## Methods

We performed a retrospective observational study in primary health care. A random sample (n = 400) of all children born between 01-01-1985 and 31-12-1988, attending school doctors between 1998 and 2000 in Leiden and Alphen aan den Rijn (located in the northern part of the province Zuid-Holland, at the western side of the Netherlands) was drawn from files of the regional public health organization (every 15^th ^file). The DCG was believed to be well known to the health personnel during the study period. There were no exclusion criteria.

The following baseline data and other observations, collected by the well-baby clinics and the school health services, were obtained from the medical records: date of birth; sex; height of parents; date and outcome of the different measurements of height and weight of the child since birth; presence of dysmorphic features or disproportion; birth weight and length, gestational age; and information about referral(s) to a specialist (date and reason). If ethnicity was not recorded, it was estimated based on the patient's first and family name according to an algorithm reported earlier [8]. If health records were incomplete, a short questionnaire was sent to all children in 2004 to obtain additional data on medical history, parental height and current height.

Three sets of referral rules were tested on the growth data: the auxological criteria mentioned in the DCG [3], those of the UKCG [5] and the cut-off values mentioned in the WHO Global Database on Child growth and Malnutrition [9]. While the UKCG concentrates only on one auxological referral criterion (Height < -2.67 SDS (= 0.4th centile) at the age of 5 years [5]), the DCG [3] focuses on several referral criteria : Height in standard deviation score (SDS), Clinical symptoms (indications of psychosocial growth retardation, dysmorfic or disproptionate features), persistent short stature after born SGA (small for gestational age), HSDScor (the distance between height SDS and the target height SDS (the estimated final height SDS of a boy or a girl on the basis of their genetic potential), and growth deflection (a downward movement of height SDS) (table 1). Target height (TH) for a boy is [father's height + (mother's height + 13)]/2 + 4.5 cm and for a girl: [mother's height + (father's height - 13)]/2 +4.5 cm, wherein 13 cm is the mean difference between final height of males and females, and 4.5 cm is the mean secular trend in one generation of 30 years. Target height can also be expressed as SDS (Z-score), by taking the difference between TH and the mean final height of a young adult of the same sex, and then divide by the SD at that age. By correcting for secular trend, the actual population reference diagrams can be used for calculating TH SDS.

**Table 1:** Auxological referral criteria taken from the Dutch Consensus Guideline [3]

***Description rule***		***Criteria***	***Rule nr*.**
Absolute height		HSDS* < -2.5 (P0.6)	1
Clinical symptoms		HSDS* < -1.3 (P10) AND (dysmorphic features OR disproportions)	2
Persistent short stature after born SGA**		SGA** AND HSDS* < -1.88 (P3) after the age of 2 years	3
HSDS_cor _^†^	♂: < 10 yr and > 13.4 yr;	HSDS* < -1.3 AND HSDS-THSDS < -1.3	4
	♀: < 9 yr and > 12.3 yr		
	Pubertal age^▪^:	HSDS* < -1.3 AND HSDS-THSDS^§ ^< -1.3 AND pubertal signs	5
	♂: 10 – 13.4 yr;		
	♀: 9 – 12.3 yr		
Deflection^‡^	♂: < 10 yr and > 13.4 yr;	T2 – T1 > 1	6a
	♀: < 9 yr and > 12.3 yr	(SDS1 – SDS2)/(T2-T1) < -0.25	
		T2 – T1 > 1	6b
		(SDS1 – SDS2) < -1	
	Pubertal age^▪^:	T2 – T1 > 1	7a
	♂: 10 – 13.4 yr;	(SDS1 – SDS2)/(T2-T1) < -0.25	
	♀: 9 – 12.3 yr	With pubertal signs	
		T2 – T1 > 1	7b
		(SDS1 – SDS2) < -1	
		With pubertal signs	

* HSDS = Height Standard Deviation Score

**SGA = Small for Gestational Age

^§ ^THSDS = Target Height Standard Deviation Score

^† ^HSDS_cor _= HSDS corrected for parental height

^‡ ^Deflection: Deflection is divided into a deflection per time interval (6a and 7a) and an absolute deflection (7b) The deflection per time interval represents a downward movement of HSDS (HSDS2-HSDS1) over time (T2-T1), while the absolute deflection is defined by a decrease of HSDS over an unspecified time period. In the categories 3–10 and 10–18, T1 > = 3 years, in the other categories T1 > 0.

^▪ ^Pubertal age: If a child does not show any pubertal signs (♂: genitals > = Tanner stage 2 OR testis volume > = 4 ml; ♀: breast > = Tanner stage 2) at this age referral is not necessary.

The WHO Global Database on Child growth and Malnutrition uses a cut-off point of -2 SD (= 2.3^rd ^centile) to classify stunting (low height for age) and underweight at all ages [9].

Because of the instability in growth pattern under the age of three years and the varying growth patterns in puberty caused by differences in pubertal timing, we decided to evaluate the guidelines in different age groups (0–3, 3–10 and 10–18 years), as well as over the whole age range. As it was hypothesized that in the age group 0–3 years a height deflection in standard deviations scores (SDS) away from the target height SDS might be a good reason to refer, we decided to test this rule (| HSDS (x_2_)- TH SDS | > | HSDS(x_1_) – TH SDS |) for both a delta HSDS (HSDS (x_2_)-HSDS(x_1_)) of -0.5 or -1 with x_2 _> x_1_.

All data were analysed in SPSS version 11 and S-plus version 7.0. Length (up to 2 years), height (from 2 years), weight and target height were expressed as standard deviation score (SDS), using the Dutch reference growth data for children of Dutch origin, children of Turkish origin, and children originating from Morocco, respectively [10-12].

HSDS (Height SDS) is the distance between the individual's height and the population's mean height for age and sex, divided by the standard deviation in the population for the same age and sex: [Individual's height - mean population's height for the same age and sex]/population's SD for the same age and sex.

A SDS can be easily converted to a percentile, using standard statistical tables. For growth charts SD lines are more suitable than percentiles, a.o. because of the the equidistance between the SDS lines. In the Dutch growth charts (Frediks 2000) -2.5, -2, -1, 0, +1, +2, and +2.5 lines are drawn, corresponding with P0.6, P2.3, P16, P50, P84, P 97.7, P99.4.

In preterm infants (gestational age < 37 weeks) length and weight SDS were corrected for gestational age. The intrauterine growth curve of the Swedish reference population [13] was used to express SDS till the age corresponding with 40 weeks of gestation. Between 40 and 42 weeks an interpolation between the growth curve of the Swedish reference population and that of the Dutch reference population was used. From 42 weeks of gestation till the age of 2 years, SDS was calculated for age corrected for gestation, using the Dutch reference growth data.

Parental height was missing in 53% of the children. We imputed these data under the assumption that the data were missing at random using Multivariate Imputation by Chained Equations (MICE) [14]. The method created multivariate imputations by applying sequential linear regressions, where each incomplete variable was imputed conditionally on all variables in an iterative fashion. The imputation model consisted of the height SDS, weight SDS, Body-Mass-Index SDS, age, gender, the height of the other parent, ethnicity, yes or no use of medication influencing growth, and place of attendance of the school doctors. The number of iterations was set to 15. Predictive mean matching was used to create parental height imputations. The imputation method includes parameter uncertainty, preserves the multivariate structure in the data and has good coverage properties [15].

Small for gestational age (SGA) was defined as a birth weight and/or length SDS < -2, comparing the present birth weights and lengths with gestational age-matched reference values from Niklasson et al [13].

The study was approved by the Medical Ethical Committee of the Leiden University Medical Center and the Regional Public Health Service Hollands Midden.

## Results

From the initial 400 children, 8 were excluded from the analyses because of incomplete growth data (for example no date of measurement). The general characteristics of the remaining 392 children are illustrated in table 2.

**Table 2:** General characteristics of the study sample

		**Total sample**
		**n = 392**
**Gender Male: n (%)**		199 (50.8)
**Ethnicity: n (%)**	**Dutch/European**	334 (85.2)
	**Turkish**	7 (1.8)
	**Moroccan**	7 (1.8)
	**Others:**	44 (11.3)
**Dysmorphic features: n (%)**		16 (4.1)
**Disproportion: n (%)**		18 (4.6)
**Target height SDS: Mean (SD)**		0.18 (0.86)
**Number of measurements (median)**		10

Table 3 shows the percentage of referrals in different age groups, which would have taken place if the Dutch consensus guideline (DCG) had been followed. Almost 80% of the sample would have been referred at some age between 0–18 years. Most referrals would take place in the first 3 years (73.9%); The first height deflection referral rule (refer if length or height SDS changes more than 0.25 SD per year) would lead to most referrals (69%), followed by the second height deflection referral rule (refer if height SDS decreases by more than 1 SD) leading to another unacceptably high number of referrals (34%). Also the referral rule based on distance to target height SDS (refer if the child's height SDS shows a distance of more than 1.3 SD to target height SDS) would lead to a high percentage of referrals (15%). In the other age groups, especially the deflection rules would also be responsible for the majority of referrals.

**Table 3:** Estimated percentage of referrals according to the DCG

***Description rule***		***Rule nr*.**	***% referrals***	***% referrals***	***% referrals***	***% referrals***
			***0–3 years***	***3–10 year***	***10–18 year***	***0–18 years***
			***N = 330***	***N = 361***	***N = 345***	***N = 392***
Absolute height		1	1.8	0.8	1.4	3.3
Clinical symptoms		2	2.4	1.1	0.9	2.3
Persistent short stature after born SGA		3	0.0	0.0	0.0	0.0
HSDS_cor_	♂: < 10 yr and > 13.4 yr;					
	♀: < 9 yr and > 12.3 yr	4	15.2	5.0	4.9	16.8
	♂: 10 – 13.4 yr;					
	♀: 9 – 12.3 yr	5	n.a.	n.a.	2.0 (2.0)*	2.0 (2.0)*
Deflection*	♂: < 10 yr and > 13.4 yr;					
	♀: < 9 yr and > 12.3 yr	6b	34.2	6.4	15.4	50.5
	♂: 10 – 13.4 yr;	7a	n.a.	n.a.	3.8 (1.4)*	4.6 (1.3)
	♀: 9 – 12.3 yr	7b	n.a.	n.a.	22.6 (5.5)	23.0 (4.8)
Total percentage of referrals			73.9	26.0	39.1	79.6

* Data on stage of puberty were frequently missing. Therefore we assumed children with missing data were in puberty at the reference pubertal age-period. The number between brackets however represents the exact percentage of referrals in the pubertal age-period, without the assumption.

† n.a = not applicable

Table 4 shows the percentage of referrals that had occurred if the UKCG or the WHO criteria would have been used. According to the UKCG only one child (0.3%) would have been referred, while 9–10% would have been referred according to the WHO. Like the DCG the criteria of the WHO are mainly met under the age of three years.

**Table 4:** Estimated percentage of referrals according to the UKCG, the WHO guideline, and a parental height deflection rule

***Description rule***	***Criteria***	***% referrals***	***% referrals***	***% referrals***	***% referrals***
		***0–3 years***	***3–10 years***	***10–18 years***	***0–18 years***
UKCG	HSDS^§ ^< -2.67 (P0.4) at age 5	n.a.	0.3	n.a	0.3
Low height for age (HSDS)^‡^	HSDS^§ ^< - 2 (P2.3)	7.3	3.6	3.2	9.2
Low weight for age (WSDS)^‡^	WSDS** < -2 (P2.3)	10.0	2.5	3.2	10.5
Parental height deflection rule (0.5 SDS)	(HSDS2^§ ^- HSDS1^§^) < -0.5, AND				
	| HSDS2^§ ^– TH SDS^† ^| > | HSDS1^§ ^– TH SDS^† ^|	58.8	18.3	28.7	62.0
Parental height deflection rule (1 SDS)	(HSDS2^§ ^– HSDS1^§^) < -1, AND				
	| HSDS2^§ ^– TH SDS^† ^| > | HSDS1 – TH SDS^† ^|	30.0	3.9	11.6	41.1

* UKCG

^§ ^HSDS = Height Standard Deviation Score

**WSDS = Weight Standard deviation score

^†^THSDS = Target Height Standard Deviation Score

^‡ ^Rules described by WHO Global database on Child growth and malnutrition.

n.a. = not applicable

Theoretically one could imagine that a deflection away from the target height SDS in the first 2–3 years of life could be a suitable referral rule. In this sample, however, these adapted deflection rules would lead to high percentages of referrals (table 4).

With respect to the actual number of referrals, 34 children were subject to extra visits to the well-baby clinic because of growth-retardation. Only one child was actually referred to a specialised centre for further diagnosis. This child had a deflection of length before the age of 1 year and was diagnosed with transient growth retardation due to dyspepsia.

## Discussion

We have confirmed the results of an earlier report in showing that implementing the Dutch consensus guideline for growth monitoring would lead to a high number of referrals, particularly before the age of 3 years. The actual number of referrals that was found in practice was just 1 out of 392 cases, so evidently the proposed guidelines were (fortunately) not properly followed. The specificity of the UK Consensus guideline (limited to one cut-off criterion, i.e. a height < -2.66 SDS at 5 years of age, i.e. the 0.4^th ^centile) would be better. On the other hand in an earlier paper we showed that the sensitivity of a height SDS cut-off to detect Turner syndrome is lower than that of a cut-off of the distance to target height [7]. The WHO guideline, as well as two additional criteria with respect to height deflection away from the target height, would lead to too many referrals.

To estimate the percentage of pathological conditions presenting with short stature and/or growth failure without further clinical symptoms or signs, one can refer to several studies. In the Wessex growth study 180 children (1.25%) in whom height on screening at school entry was on or below the 3^rd ^percentile, were further examined [16]. Among this group 8 children (4.4%) were newly identified as having an organic disease. Ahmed et al reported in the Oxford study 7 newly recognized children (3.0%) with organic disease among the 260 children (1.3% of all screened children) whose height was below 2 SDS, measured at the ages of 3 and 4.5 years [17]. From the 555 children (0.5% of the screened population) who were examined for their poor growth (height below the 3^rd ^percentile and/or growth rate below 5 cm/yr) in the Utah growth study 25 children (4.5%) were newly discovered as having GHD, hypothyroidism or Turner syndrome, and another 53 children (9.5%) had other medical reasons [18]. So, one can conclude that out of the (by definition) 2.3% short children (height < -2 SDS) in the population, 3–14% have a condition that warrants additional diagnostic tests, corresponding with 0.07 to 0.32% of the population. This low prevalence of pathology in children presenting with short stature implies that the specificity of the referral rules should be high, in the order of 98–99%, in order to keep the number of unnecessary referrals acceptable.

This study confirmed and expanded the earlier analysis by Van Buuren et al of the consequences of following the DCG [4]. In the earlier study the overall referral percentage until the age of 10 years was 38% (without deflection 0–3 years) or over 84% (including deflection 0–3 years). The present study found that almost 80% would have been referred in the age range 0–18 years.

Short stature has many causes, and the relative prevalence of these causes varies considerably in the world. This implies that the objectives of growth monitoring programs heavily depend on the setting. In developing countries the principal aim of growth monitoring is to detect malnutrition. Given this aim the WHO promotes growth monitoring and research to improve the monitoring procedures [9,19]. In industrialized countries, however, where there is less malnutrition, growth monitoring is aimed at detecting disorders associated with growth retardation without other clinical symptoms or signs, of which Turner syndrome, Growth Hormone deficiency and Celiac Disease are most prevalent. We have now shown that, given the low prevalence of such pathology (0.07 to 0.32% of the population), also the WHO-criteria would lead to too many referrals.

Most experienced clinicians use three archetypal criteria in the analysis of growth: the distance of the child's height to the mean of the population (either expressed as centile or as SD score), the distance between height SDS and target height (the gender-corrected mid-parental height) and growth retardation (i.e. deflection of the growth curve across the SD-lines or centiles). Our study confirmed that height SDS deflection over time ("slow deflection")leads to far too many referrals. In earlier studies we have shown that height SDS deflection is a poor predictor of congenital growth hormone deficiency [20] and Turner syndrome [7]. On the other hand, clinical experience teaches that crossing the SD-lines (or centiles) can be the first sign of an acquired GH deficiency (e.g. caused by a brain tumor), Cushing syndrome or hypothyroidism. The distance to target height is the best decision rule to detect children with Turner syndrome, one of the main causes of short stature [7], but it was left out of the UKCG because of practical and theoretical problems. For an optimally efficacious and efficient growth monitoring algorithm, a combination of the three main criteria should be sought.

## Conclusion

We have confirmed that the current Dutch consensus guideline would lead to too many referrals, mainly due to the deflection of length during the first 3 years of life. The UKCG leads to far less referrals, but may be insensitive to detect Turner syndrome, and probably other clinically relevant growth disorders. New guidelines for growth monitoring are needed, which combine a low percentage of false positive results with a good sensitivity. To achieve these goals more studies are needed on the diagnostic value and cost-effectiveness of auxological screening for the diagnosis of various diseases.

## Competing interests

The author(s) declare that they have no competing interests.

## Authors' contributions

All authors contributed to the planning, the design of the study and read and approved the final manuscript. FKG and AL were responsible for the collection of the data. All the other authors participated together with FKG in the analytical part. FKG wrote the manuscript.

## Pre-publication history

The pre-publication history for this paper can be accessed here:


